# Luteal blood flow as a predictive factor for methotrexate treatment outcomes in women with unruptured tubal pregnancy

**DOI:** 10.1186/s12884-020-02882-3

**Published:** 2020-03-30

**Authors:** Li Wang, Meili Pei, Ting Yang, Juan Zhao, Xiaofeng Yang

**Affiliations:** Department of Gynecology and Obstetrics, The First Affiliated Hospital of Xi’an Jiaotong University, Xi’an, Shaanxi People’s Republic of China

**Keywords:** Luteal blood flow, Methotrexate, Unruptured tubal pregnancy, Predictive factor

## Abstract

**Background:**

Blood flow in the corpus luteum is associated with luteal function. However, the impact of luteal blood flow on methotrexate (MTX) treatment in women with unruptured tubal pregnancy has not been reported. The aim of the present study was to observe the impact of luteal blood flow on the therapeutic effect of MTX in women with unruptured tubal pregnancy.

**Methods:**

A prospective observational study recruited 129 women with unruptured tubal pregnancy in the First Affiliated Hospital of Xi’an Jiaotong University from September 2016 to June 2018. One hundred and fifteen women were treated successfully with MTX, and women were divided into 2 groups according to luteal blood flow: the poor luteal blood flow group and the abundant luteal blood flow group. The therapeutic effects were compared between the two groups.

**Results:**

Women in the abundant luteal blood flow group had a significantly higher serum β-human chorionic gonadotropin (β-hCG) level 4 days, 1 week and 2 weeks after MTX treatment compared with women in the poor luteal blood flow group (*P* < 0.05). The average diameter of the ectopic mass 1 week, 2 weeks and 3 weeks after MTX treatment in women with abundant luteal blood flow was significantly larger (*P* < 0.05), and the time of serum β-hCG clearance and ectopic mass disappearance were significantly longer compared with those in women in the poor luteal blood flow group (*P* < 0.05).

**Conclusions:**

Luteal blood flow might be a predictive factor for MTX treatment outcomes in women with unruptured tubal pregnancy, and those with abundant luteal blood flow need a longer recovery time.

## Background

Ectopic pregnancy (EP) occurs when blastocysts are implanted outside the uterine cavity, and EP may result in severe complications, including rupture of the fallopian tube, hemorrhagic shock, and even death [1, 2]. It has been reported that the incidence of EP represents 1–2% of all pregnancies and is the main reason of death in the first trimester of pregnancy [3]. Fallopian tubes are the most common site for blastocyst implantation [4]. At present, most EPs can be diagnosed in the early stage because of the improvement and popularization of transvaginal ultrasound (TVUS) [5]. Once a definitive diagnosis of EP has been made, the patient should be monitored closely and treated with surgical, medical or expectant management. The selection of the therapeutic plan depends on the serum human chorionic gonadotropin (hCG) level, size of the ectopic mass and foetal cardiac activity (FCA). Methotrexate (MTX) is widely used as a first-line treatment for women diagnosed with unruptured EP with a low serum hCG concentration, a small ectopic mass and stable haemodynamics. In appropriately selected women, the success rates of single-dose MTX for tubal EP treatment ranged from 65 to 95%, and 3 to 27% of the patients needed a second dose [6].

The blood flow in the corpus luteum can be detected by transvaginal colour-pulsed Doppler ultrasonography. The luteal blood flow increases gradually after ovulation, and the corpus luteum is filled with blood [7]. Previous studies have found that insufficiency of the corpus luteum during pregnancy was associated with infertility and spontaneous abortion in the first trimester of pregnancy [8, 9]. In addition, blood flow in the corpus luteum is closely related to luteal function. Some drug treatments could improve corpus luteum function by increasing its blood supply [10]. However, to the best of our knowledge, the impact of luteal blood flow on MTX treatment in women with unruptured tubal pregnancy has not been reported. Our research aims to observe the impact of luteal blood flow on MTX treatment outcomes in women with unruptured tubal pregnancy.

## Methods

### Study design and participants

A total of 129 women with unruptured tubal pregnancy in the First Affiliated Hospital of Xi’an Jiaotong University from September 2016 to June 2018 were recruited for this prospective observational study. The women had a median age of 32.8 years with an age range of 21–39 years. The diagnosis of tubal EP was based on the serum β-hCG level, lack of intrauterine gestational sac and existence of an ectopic mass on the side of the ovary, which was checked with TVUS. All participants provided written informed consent according to procedures approved by the Ethics Committee of The First Affiliated Hospital of Medical College of Xi’an Jiaotong University.

The inclusion criteria included women who were diagnosed with tubal EP and had a β-hCG level lower than 3000 mIU/mL before treatment without FCA and an ectopic pregnancy size < 4 cm. The exclusion criteria included EP at other locations and contraindications for MTX treatment, including renal and hepatic disease, active pulmonary disease, immunodeficiency and peptic ulcer [11]. Demographic data such as age, body mass index (BMI), number of pregnancies and deliveries, pregnancy method, risk factors for EP, duration of pregnancy, and clinical symptoms such as abdominal pain and vaginal bleeding were recorded. The serum β-hCG and progesterone concentrations were detected in our hospital laboratory by chemiluminescence methods. Women were checked by TVUS before treatment, and findings such as the size of the ectopic mass, yolk sac and free fluid in the Douglas cavity were recorded.

**Fig. 1:**
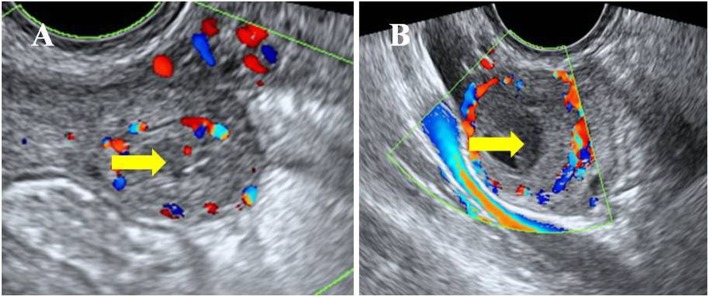
Luteal blood flow verified by TVUS. The arrow indicates the corpus luteum. **a** Poor blood flow: linear or stellate blood flow around the corpus luteum. **b** Abundant blood flow: hemicycle or annular blood flow around the corpus luteum

All ultrasound scans were performed using a Voluson E8 (GE Medical Systems, USA) ultrasound system with a vaginal probe frequency of 4–9 MHz. Blood flow was identified in the peripheral area of the corpus luteum, and women were divided into 2 groups according to the corpus luteal blood flow patterns: the poor luteal blood flow group and the abundant luteal blood flow group (Fig. 1).

Three-dimensional power Doppler ultrasound is generally superior to colour Doppler imaging for detecting low-velocity flows and visualizing small vessels. To further detect the index of the luteal blood flow, the ultrasound instrument was switched to 3D mode with power Doppler. The vascularization indices were determined using the VOCAL (virtual organ computer-aided analysis) imaging program to measure the volume, vascularization index (VI), flow index (FI), and vascularization flow index (VFI) of the corpus luteum before treatment.

### Outcome measures

All women received intramuscular MTX at a dose of 50 mg/m^2^ of body-surface area [12]. The side effects of MTX were assessed and recorded. According to the protocol, the serum β-hCG level was measured 4 days after MTX treatment, and then serum β-hCG measurements were performed weekly after MTX treatment until β-hCG was no longer detectable. In addition, the size of the ectopic mass was monitored through TVUS weekly after treatment until it disappeared. If the β-hCG level failed to decline by at least 15% between days 4 and 7, then a second dose of MTX was given [6]. Whenever haemodynamic instability and/or clinical symptoms of tubal rupture (i.e., increased abdominal pain in combination with a decline in the haemoglobin level and signs of pelvic haemorrhage) occurred, surgical intervention was performed.

The primary outcome was the decline in serum β-hCG to an undetectable level. Secondary outcomes included the serum β-hCG clearance time, reduction and clearance time of the ectopic mass, and the side effects of MTX treatment (i.e., nausea, vomiting, diarrhoea, and elevation of the transaminase level).

### Statistical analysis

The success rates of MTX for tubal ectopic pregnancy ranged from 60 to 90% according to previous studies [6]. To achieve 80% power with a 2-sided alpha of 0.05, we needed to recruit 116 women in the study. Considering a loss to follow-up of 5–10%, we set an overall recruitment goal of 129 women. Statistical analyses were performed using SPSS version 20.0 (IBM, Armonk, NY, USA). The Kolmogorov–Smirnov test was used to check for normal distribution prior to statistical tests. Normally distributed continuous variables were represented as mean ± standard deviation, and skewed data were represented as median (25, 75% quartile range). The non-parametric Mann–Whitney *U* test was used to analyze the continuous variables. Differences in dichotomous outcomes between the two groups were represented as number and percentage (%), which were compared by chi-square test or Fisher’s exact test when the expected frequencies fell below five. *P* < 0.05 was considered statistically significant.

## Results

**Fig. 2:**
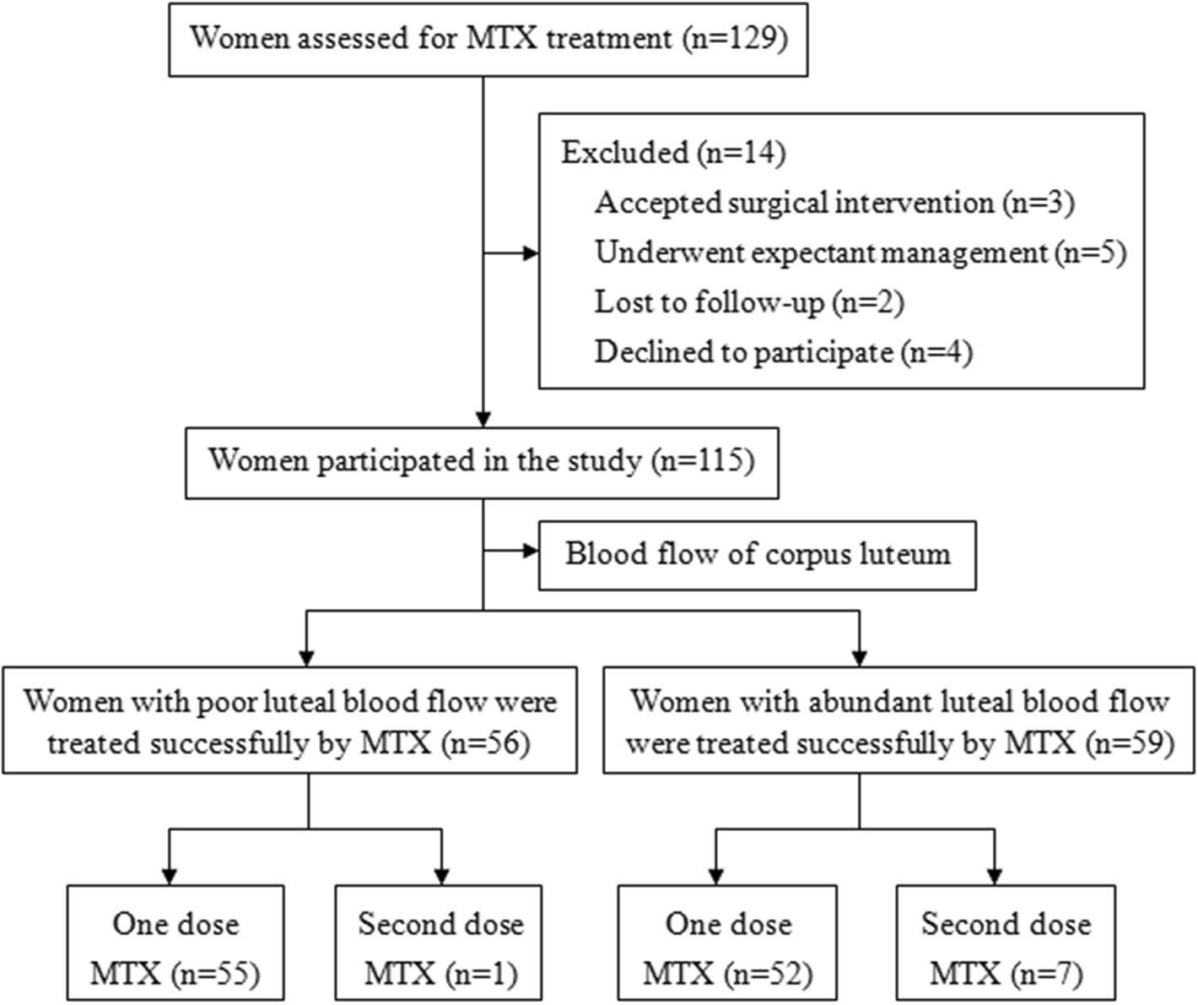
Flow chart of study participation

A total of 129 women were assessed for MTX treatment, and of these, we excluded 3 women who underwent surgical intervention, 5 women who underwent expectant management, 2 women who were lost to follow-up, and 4 women who declined to participate. Finally, 115 women were included in the study, and these women were divided into 2 groups according to the luteal blood flow. Fifty-six women were successfully treated with MTX in the poor luteal blood flow group, and 59 women were successfully treated with MTX in the abundant luteal blood flow group (Fig. 2).

Table 1 illustrates the baseline characteristics of the women in the two groups. No significant difference was found when comparing maternal age, BMI, number of pregnancies and deliveries, pregnancy method, risk factors of ectopic pregnancy, duration of pregnancy, clinical symptoms, serum β-hCG level before treatment, or ultrasound findings (*P* > 0.05). However, the serum progesterone level before treatment was significantly higher in women with abundant luteal blood flow than in those with poor luteal blood flow (*P* = 0.041).

**Table 1:** Baseline characteristics of women in the two groups

Characteristics	Poor blood flow group (*n* = 56)	Abundant blood flow group (*n* = 59)	*P*-value^a^
Maternal age (years)	32.4 ± 7.9	33.1 ± 9.3	0.413
BMI (kg/m^2^)	22.5 ± 4.9	23.2 ± 5.0	0.769
Number of pregnancies	2.2 ± 0.8	2.2 ± 0.7	0.802
Number of deliveries	1.0 ± 0.4	0.9 ± 0.5	0.613
Pregnancy method			0.866
Natural conception	49 (87.5)	51 (86.4)	
ART	7 (12.5)	8 (13.6)	
Risk factors of EP			
Previous EP	3 (5.4)	5 (8.5)	0.511
Pregnant with IUD	4 (7.1)	3 (5.1)	0.645
Duration of pregnancy (days)	46.6 ± 7.1	48.3 ± 8.2	0.598
Clinical symptoms			
Abdominal pain	47 (83.9)	48 (81.4)	0.716
Vaginal bleeding	51 (91.1)	54 (91.5)	0.931
Pretreatment β-hCG (mIU/mL)	1365.6(769.2–2824.5)	1403.2(813.1–2902.5)	0.497
Pretreatment progesterone (nmol/L)	26.7(15.8–46.9)	42.3(23.7–71.5)	0.041
Ultrasound findings			
Average diameter of ectopic mass (cm)	2.1(1.3–3.2)	2.3(1.6–3.5)	0.764
Yolk sac	11 (19.6)	14 (23.7)	0.928
Free fluid in Douglas cavity	44 (78.6)	46 (78.0)	0.937

Values are presented as mean ± SD, median (quartile range) or number (percentage)

*BMI* Body mass index, *ART* Assisted reproductive technology, *EP* Ectopic pregnancy, *IUD* Intrauterine device

^a^ Mann-Whitney *U* test, Chi-square test or Fisher’s exact test

**Fig. 3:**
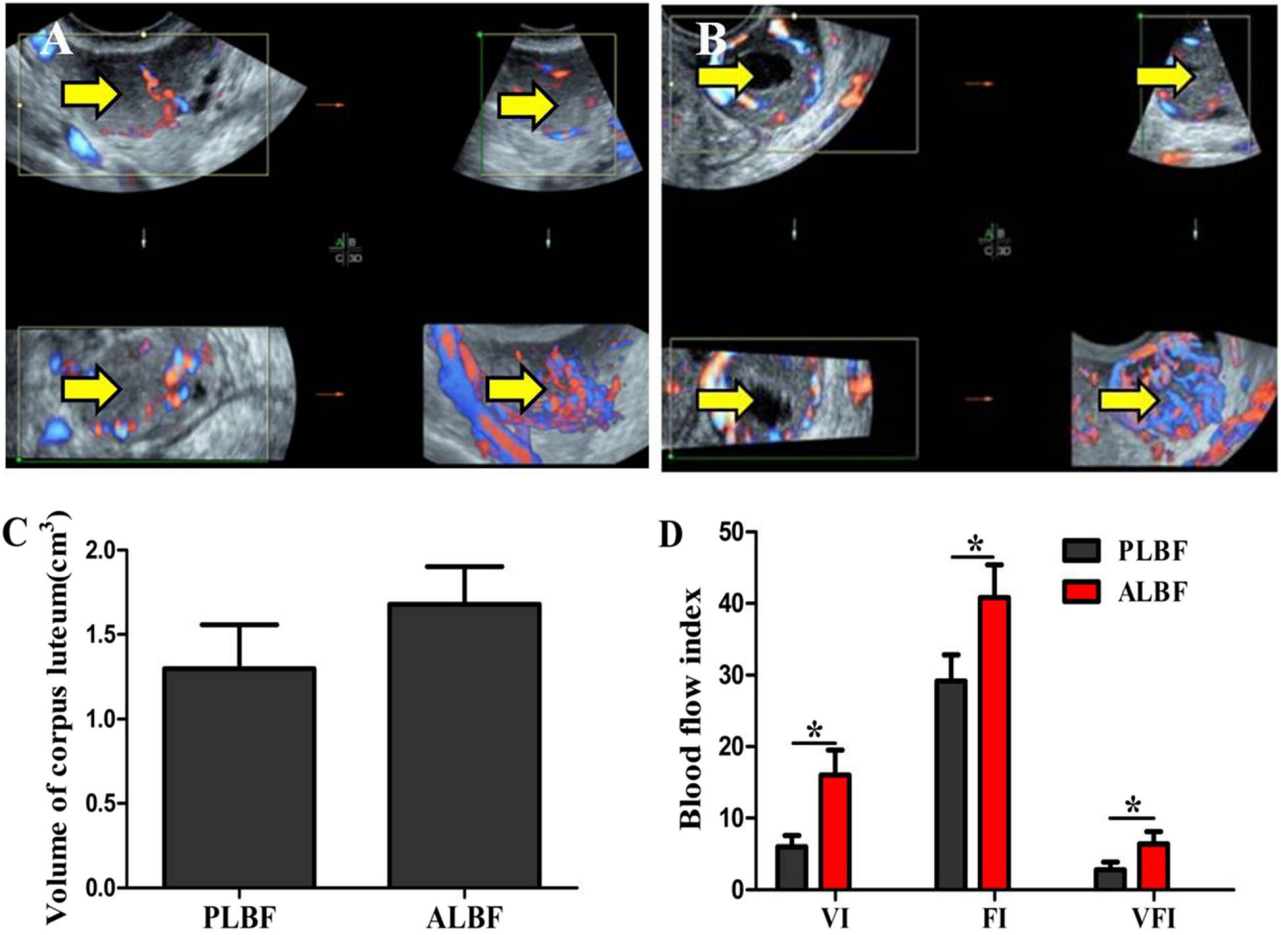
Corpus luteum measured by 3D power Doppler ultrasound. The arrow indicates the corpus luteum (*P < 0.05). PLBF: Poor luteal blood flow group, ALBF: Abundant luteal blood flow group. **a** Poor luteal blood flow and **b** abundant luteal blood flow are presented. **c** and **d** Comparison of volume, VI, FI and VFI of the corpus luteum measured by 3D power Doppler ultrasound between PLBF and ALBF

No significant difference was found when comparing the volume of the corpus luteum (*P* = 0.058); however, the VI (*P* = 0.019), FI (*P* = 0.042) and VFI (*P* = 0.031) of the corpus luteum were significantly higher in women with abundant luteal blood flow than in women with poor luteal blood flow (Fig. 3).

**Fig. 4:**
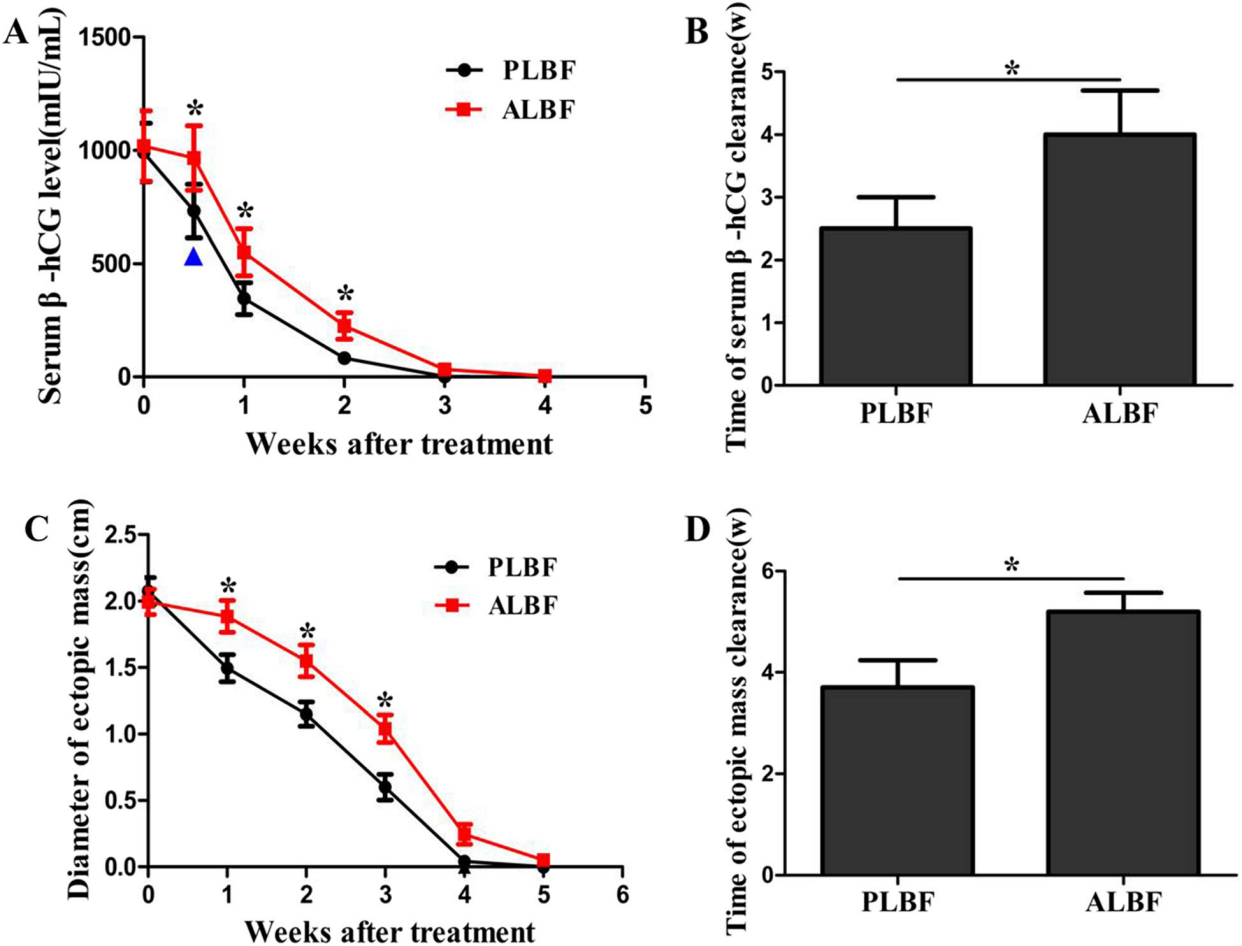
Therapeutic effect of MTX treatment during follow-up. ^▲^: 4 days after MTX treatment (*P < 0.05). PLBF: Poor luteal blood flow group, ALBF: Abundant luteal blood flow group. **a** Serum β-hCG levels at different times after treatment. **b** The time required for serum β-hCG clearance. **c** The average diameter of the ectopic mass at different times after treatment. **d** The time required for ectopic mass disappearance

The serum β-hCG level 4 days, 1 week and 2 weeks after MTX treatment in women with abundant luteal blood flow were significantly higher than those in women in the poor luteal blood flow group (*P* < 0.05). Moreover, the time of serum β-hCG clearance in women with abundant luteal blood flow was significantly longer (*P* = 0.029). The average diameter of the ectopic mass 1 week, 2 weeks and 3 weeks after MTX treatment in women with abundant luteal blood flow was significantly larger (*P* < 0.05), and the time required for ectopic mass disappearance was significantly longer (*P* = 0.043) compared with that in women in the poor luteal blood flow group (Fig. 4).

In women with poor luteal blood flow, 55 women (98.2%) received one dose of MTX treatment, and 1 woman (1.8%) received a second dose of MTX treatment because her serum β-hCG level did not decline by 15% between days 4 and 7. However, in women with abundant luteal blood flow, 52 women (88.1%) received one dose of MTX treatment, and 7 women (11.9%) received a second dose of MTX treatment. There was a significant difference in the dose of MTX used in the two groups (*P* = 0.034). Eight women with poor luteal blood flow reported side effects versus 11 in the abundant luteal blood flow group, and the laboratory results showed a minor rise in alanine aminotransferase (ALT) and aspartate transaminase (AST) levels, which ranged from 45 to 64 U/L. These side effects were mild and disappeared within 2 weeks after MTX treatment. No significant difference was found when comparing the side effects of MTX between the two groups (*P* = 0.529) (Table 2).

**Table 2:** Dose and side effects of MTX treatment

Variable	Poor blood flow group (*n* = 56)	Abundant blood flow group (*n* = 59)	*P*-value^a^
Success rate of MTX treatment			0.034
One dose	55 (98.2)	52 (88.1)	
Second dose ^b^	1 (1.8)	7 (11.9)	
Side effects of MTX	8 (14.3)	11 (18.6)	0.529
Clinical symptoms			
Nausea	3 (5.4)	5 (8.5)	
Diarrhoea	2 (3.6)	2 (3.4)	
Laboratory results			
Rise of ALT	2 (3.6)	3 (5.1)	
Rise of AST	1 (1.8)	1 (1.7)	

Values are presented as number (percentage)

*ALT* alanine aminotransferase, *AST* aspartate transarninase

^a^Chi-square test or Fisher’s exact test

^b^Second dose was given if serum β-hCG level did not decline 15% between days 4 and 7

## Discussion

Since the first application by Tanaka et al. [13], MTX has become the primary treatment for unruptured EP [14]. The successful treatment of EP depends on several factors, including the serum hCG level, route of MTX administration, size of the ectopic mass and FCA [15–17]. However, the effect of luteal blood flow during pregnancy on MTX treatment in women with EP has not been previously reported. Transvaginal colour Doppler ultrasound is a useful and noninvasive technique for the detection of blood flow around the corpus luteum [18]. Previous studies reported that luteal blood flow was associated with luteal vascularization and luteal function [19, 20], which is essential for the support of early pregnancy. Therefore, we speculate that the blood supply of the corpus luteum may be related to the treatment outcomes of unruptured tubal pregnancy.

The present study observed 115 women with unruptured tubal pregnancy who were treated successfully with MTX. Women in the study were divided into 2 groups according to their luteal blood flow. Our data revealed that women with abundant luteal blood flow had a significantly higher serum β-hCG level 4 days, 1 week and 2 weeks after MTX treatment compared with women with poor luteal blood flow. Moreover, the average diameter of the ectopic mass 1 week, 2 weeks and 3 weeks after MTX treatment in women with abundant luteal blood flow was significantly larger, and the time required for serum β-hCG clearance and ectopic mass disappearance were significantly longer in comparison with that in women with poor luteal blood flow. These findings illustrated that luteal blood flow impacted the therapeutic outcomes in women with unruptured tubal pregnancy, and those with abundant luteal blood flow needed a second dose of MTX treatment and a longer recovery time.

Previous studies confirmed that the crucial role of angiogenesis in corpus luteum is maintenance of luteal function [21]. Ovulation is triggered through a peak of luteinizing hormone (LH) in blood, which is secreted and released by hypothalamus. The pressure in the follicular cavity decreases after ovulation, and the avascular granulosa cell layer is invaded by the vascular endothelial cells of the theca cell layer, and then the blood flow in the corpus luteum increase rapidly [22]. In fact, vascularization and abundant blood flow in the corpus luteum are important for the development of the corpus luteum and progesterone secretion, which play an important role in the improvement of endometrial receptivity, embryo implantation and successful pregnancy.

Blood vessels are maintained in the corpus luteum until 10 weeks of gestation. The function of the corpus luteum is mainly used for the secretion of progesterone and its release into the blood. Previous studies displayed that the removal of the corpus luteum in the first trimester of pregnancy leads to a decline in blood progesterone level, which further causes miscarriages [21]. Multiple studies have confirmed that the blood supply of the corpus luteum is correlated with the serum progesterone level, and the blood supply of the corpus luteum is decreased in women with luteal phase defects compared with that in women with normal luteal function [10, 18, 20]. Progesterone maintains embryonic development during pregnancy in a variety of ways, such as by increasing the excitatory threshold of uterine smooth muscle, inhibiting uterine contraction, participating in the maintenance of the maternal-foetal microenvironment, and promoting maternal-foetal tolerance [23–26]. The data in our study demonstrated that the serum progesterone level before treatment and the VI, FI and VFI of the corpus luteum were significantly higher in women with abundant luteal blood flow than those in women in the poor luteal blood flow group. These findings indicated that luteal blood flow was an important factor in regulating progesterone levels and affected luteal function and pregnancy outcomes. Low blood supply to the corpus luteum was associated with luteal function defects, which is consistent with previous reports [20].

This study has some limitations. Corpus luteum function is affected by factors other than the corpus luteal blood flow according to this study design. In addition, if an ectopic pregnancy had already been aborted due to falling β-hCG levels, this may not have had any effect on intervention with MTX. Therefore, the repeatability of the results of this study needs to be confirmed after excluding other factors that affect luteal function and therapeutic effect.

## Conclusions

In conclusion, the present study showed the impact of luteal blood flow during pregnancy on MTX treatment in women with unruptured tubal pregnancy. Those with abundant luteal blood flow needed a second dose of MTX treatment and a longer recovery time. The blood supply of the corpus luteum may be an important clinical factor for assessing the prognosis of women with EP.

## Data Availability

The datasets used and/or analyzed during the current study are available from the corresponding authors on reasonable request.

## Abbreviations

EP: Ectopic pregnancy
TVUS: Transvaginal ultrasound
hCG: Human chorionic gonadotropin
FCA: Fetal cardiac activity
MTX: Methotrexate
VI: Vascularization index
FI: Flow index
VFI: Vascularization flow index
BMI: Body mass index
ART: Assisted reproductive technology
IUD: Intrauterine device
PLBF: Poor luteal blood flow group
ALBF: Abundant luteal blood flow
ALT: Alanine aminotransferase
AST: Aspartate transarninase
